# Plasmon-Enhanced Piezo-Photocatalytic Degradation of Metronidazole Using Ag-Decorated ZnO Microtetrapods

**DOI:** 10.3390/molecules30234643

**Published:** 2025-12-03

**Authors:** Farid Orudzhev, Makhach Gadzhiev, Rashid Gyulakhmedov, Sergey Antipov, Arsen Muslimov, Valeriya Krasnova, Maksim Il’ichev, Yury Kulikov, Andrey Chistolinov, Damir Yusupov, Ivan Volchkov, Alexander Tyuftyaev, Vladimir Kanevsky

**Affiliations:** 1 Smart Materials Laboratory, Dagestan State University, M. Gadzhiev 43a, 367000 Makhachkala, Russia; raxa.keyt@gmail.com; 2 Joint Institute for High Temperatures, Russian Academy of Sciences, 125412 Moscow, Russia; 3 Complex of Crystallography and Photonics, National Research Centre “Kurchatov Institute”, 119333 Moscow, Russia

**Keywords:** zinc oxide, piezo-photocatalysis, solar radiation, microtetrapods, silver nanoparticle, piezophototronic effect, plasmon resonance, raman scaterring

## Abstract

The development of advanced semiconductor-based catalysts for the rapid degradation of emerging pharmaceutical pollutants in water remains a critical challenge in environmental science. In this study, we present the synthesis, characterization, and catalytic performance of zinc oxide (ZnO) microtetrapods decorated with plasmonic Ag nanoparticles. These microtetrapods have been designed to enhance piezo-, photo-, and piezo-photocatalytic degradation of metronidazole (MNZ), a persistent antibiotic contaminant. ZnO microtetrapods were synthesized by high-temperature pyrolysis and using atmospheric-pressure microwave nitrogen plasma, followed by photochemical deposition of Ag nanoparticles at various precursor concentrations (0–1 mmol AgNO_3_). The structural integrity of the samples was confirmed through X-ray diffraction (XRD) analysis, while the morphology was examined using scanning electron microscopy with energy-dispersive X-ray analysis (SEM-EDX). Additionally, spectroscopic analysis, including Raman, electron paramagnetic resonance (EPR), and photoluminescence (PL) spectroscopy, was conducted to verify the successful formation of heterostructures with adjustable surface loading of Ag. It has been shown that ZnO microtetrapods decorated with plasmonic Ag nanoparticles exhibit Raman-active properties. A systematic evaluation under photocatalytic, piezocatalytic, and combined piezo-photocatalytic conditions revealed a pronounced volcano-type dependence of catalytic activity on Ag content, with the 0.75 mmol composition exhibiting optimal performance. In the presence of both light irradiation and ultrasonication, the optimized Ag/ZnO composite exhibited 93% degradation of MNZ within a span of 5 min, accompanied by an apparent rate constant of 0.56 min^−1^. This value stands as a significant improvement, surpassing the degradation rate of pristine ZnO by over 24-fold. The collective identification of defect modulation, plasmon-induced charge separation, and piezoelectric polarization as the predominant mechanisms driving enhanced reactive oxygen species (ROS) generation is a significant advancement in the field. These findings underscore the synergistic interplay between plasmonic and piezoelectric effects in oxide-based heterostructures and present a promising strategy for the efficient removal of recalcitrant water pollutants using multi-field activated catalysis.

## 1. Introduction

Structures with sophisticated spatial configurations and morphologies are among the most intensively studied materials [1,2,3,4,5]. Such materials include hierarchical nanostructures, nanoporous membranes, metallic nanocarcasses, fullerenes, nanocarbon meshes, etc. Their key feature is a high surface-to-volume ratio, which makes them highly efficient in applications such as sensing, catalysis, plasmonics, and data storage. Nanostructures with intricate spatial geometries can be fabricated using chemical methods, photolithography, ion etching, and other techniques. Among these approaches, the spontaneous nucleation method occupies a special place. Although this method offers limited control over the size and morphology of the resulting nanostructures, it compensates for this drawback with high efficiency and procedural simplicity. Examples of spontaneously nucleated hierarchical nanostructures include ZnO tetrapods [6,7,8].

Overall, ZnO nanostructures, along with TiO_2_, are among the most studied catalysts in the processes of photostimulated degradation of persistent organic pollutants [9,10]. Photocatalysis employs semiconductor materials to generate electron–hole pairs under light irradiation, leading to the formation of ROS such as hydroxyl radicals (^•^OH) and superoxide anions (^•^O_2_^−^) that oxidize organic molecules.

It is worth noting that ZnO tetrapods are particularly promising catalysts, as they exhibit enhanced activity under visible-light irradiation [11]. When combined with other functional nanomaterials, ZnO tetrapods represent a valuable platform for a wide range of future applications. Owing to their ability to hyper-amplify signals in electronics and sensor devices, metal–semiconductor systems have found wide application. The amplification phenomenon arises from resonant light absorption at the metal–semiconductor boundary, leading to the excitation of localized plasmons [12,13,14]. Enhanced antibacterial and photocatalytic performance of ZnO/Au and ZnO/Ag nanocomposites has been demonstrated in Ref. [15]. Another important application of ZnO/Ag systems is the detection of ultra-low contamination levels by means of surface-enhanced Raman scattering (SERS) [16].

Currently, significant efforts are being made to enhance the photocatalytic efficiency of materials, and considerable progress has already been achieved [17,18,19]. Sonocatalysis employs ultrasound to induce cavitation, thus generating localized high temperatures and pressures that enhance ROS production, whereas piezocatalysis exploits mechanical stress to induce piezoelectric polarization and drive catalytic reactions [20,21,22]. Notably, the synergistic combination of light and mechanical activation, termed piezo-photocatalysis, has demonstrated superior performance by suppressing electron–hole recombination and enhancing ROS generation. This approach thus offers considerable advantages for degrading persistent pollutants [23].

In this context, ZnO is a unique material due to its pronounced photocatalytic and piezoelectric properties. Piezoelectric properties of ZnO originate from a non-centrosymmetric crystalline structure [24,25]. Moreover, the highest values of piezoelectric coefficients are observed in ZnO rod-like structures [26], where maximum structural relaxation and related shrinkage of the unit supercell are achieved.

The successful integration of mechanical stimulation during photo-stimulated catalysis (the piezophototronic effect) has been demonstrated in ZnO structures of various morphologies, with the maximum effect observed in rod-like structures with clearly pronounced facets [27]. By supplementing the piezocatalytic effect with plasmonic enhancement, a synergetic effect can be achieved: the independent enhancement of photocatalytic efficiency by inducted piezoelectric fields and plasmonic light absorption, as well as the ability to control the position of plasmonic nanostructures by mechanical deformation [28]. Such a concept was previously implemented in an Ag/ZnO system [29]. Despite promising results obtained by the authors, these studies were not continued further, likely due to the complexities of practical application. Specifically, ZnO nanotetrapods were used to randomly deposit Ag particles. Such nanomaterials also demonstrate rather high toxicity: as their size decreases to tens of nanometers, their ability to penetrate human organs increases significantly. While some studies report no observable harmful effect of nanoparticles on the human body [30], more complex comprehensive research is required to confirm this. In addition, nanoparticles present significant difficulties in view of their use and further disposal.

The authors of this work have extensive experience in synthesizing ZnO microstructures using various methods [31,32]. With appropriate modifications, plasma technology can be adapted to produce ZnO microtetrapods (hereinafter referred to as tetrapods) with pronounced facets on a large scale. Furthermore, modeling results [33] show the possibility to enhance the piezoelectric potential in elongated rod-like ZnO structures. Specifically, in rods longer than 600 nm, the piezoelectric potential can reach 434 mV under a cavitation pressure of 10^8^ Pa.

Metronidazole (MNZ) is a widely used antibiotic in both human and veterinary medicine whose extensive application has led to its ubiquitous presence in environmental matrices, particularly in wastewater effluents and surface waters [34,35]. Its persistence and resistance to biodegradation make MNZ a significant pharmaceutical contaminant that poses ecological risks due to its toxicity, potential mutagenicity, and role in promoting antimicrobial resistance among aquatic microorganisms [36,37]. Conventional treatment methods such as adsorption, chlorination, membrane separation, and biological processes have been extensively employed for removing pharmaceutical contaminants; however, they suffer from inherent limitations. Adsorption techniques, while capable of transferring contaminants from the aqueous phase to solid media, do not destroy the compounds and often result in secondary pollution and regeneration issues [38]. Similarly, chlorination and membrane separation have limited efficacy in degrading stable molecules like MNZ, often leading to the formation of toxic by-products and membrane fouling, respectively. Biological treatments, despite being environmentally friendly, frequently fail to completely remove non-biodegradable pharmaceuticals, particularly under conditions where useful microbial populations are compromised by the contaminants themselves. Studies show that conventional wastewater treatment plants remove MNZ only partially, requiring the use of photocatalytic treatment methods. The degradation mechanism and kinetics of MNZ in the presence of HO^•^ and SO_4_^•−^ were studied using density functional theory (DFT) [39]. Composite materials like BiTmDySbO_7_/BiEuO_3_ [40], Ag-N-SnO_2_ [41], etc., are most often used as photocatalysts in MNZ degradation processes.

The presented study investigates the morphology, crystal structure, and plasmon-enhanced piezo-photocatalytic and SERS properties of silver nanoparticle-doped zinc oxide microtetrapods as a function of silver nanoparticle content. MNZ was used as a difficult-to-oxidize organic pollutant.

## 2. Results

### 2.1. Study of Morphology, Elemental, and Structural-Phase Compositions of Ag/ZnO Tetrapods

**Figure 1:**
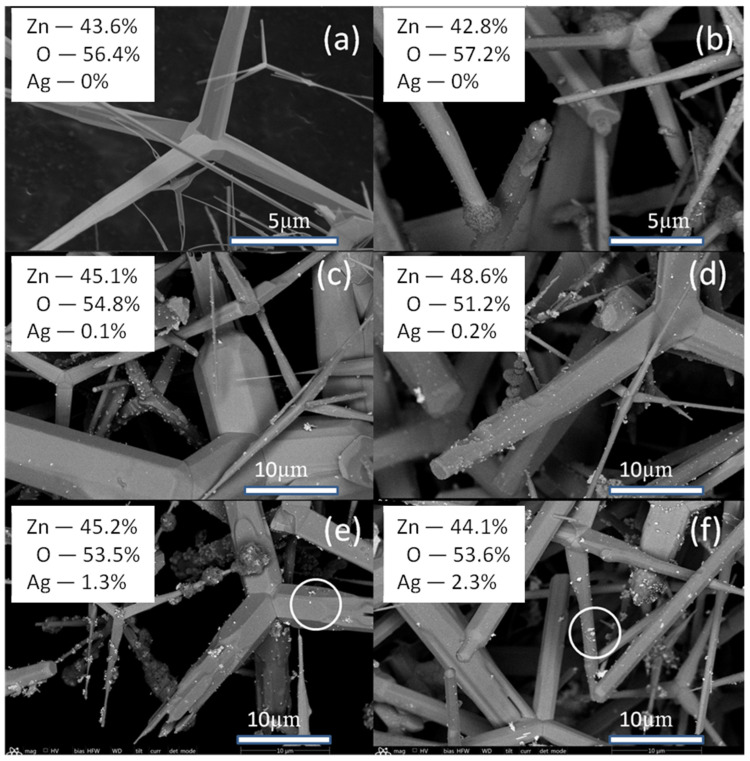
SEM images of ZnO/Ag microstructures: types 0 (**a**); 0.1 (**b**); 0.25 (**c**); 0.5 (**d**); 0.75 (**e**); 1 (**f**). Designation: white circles—clusters of Ag particles.

According to the electron microscopy data, during Ag deposition from an AgNO_3_ solution onto the surface of ZnO tetrapods (Figure 1), an island-like structure is formed. The structure comprises discrete islands measuring 100–200 nm in size, alongside larger, branched, and shapeless sub-micron formations.

**Figure 2:**
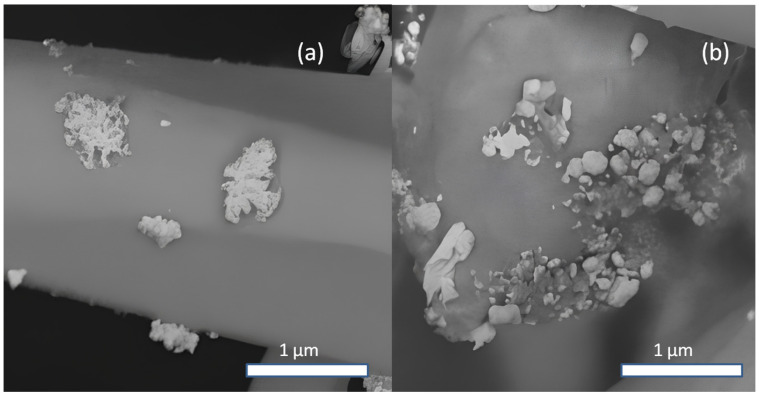
SEM images of typical Ag nanoparticle clusters on ZnO tetrapods facets: types 0.75 (**a**); 1 (**b**).

**Figure 3:**
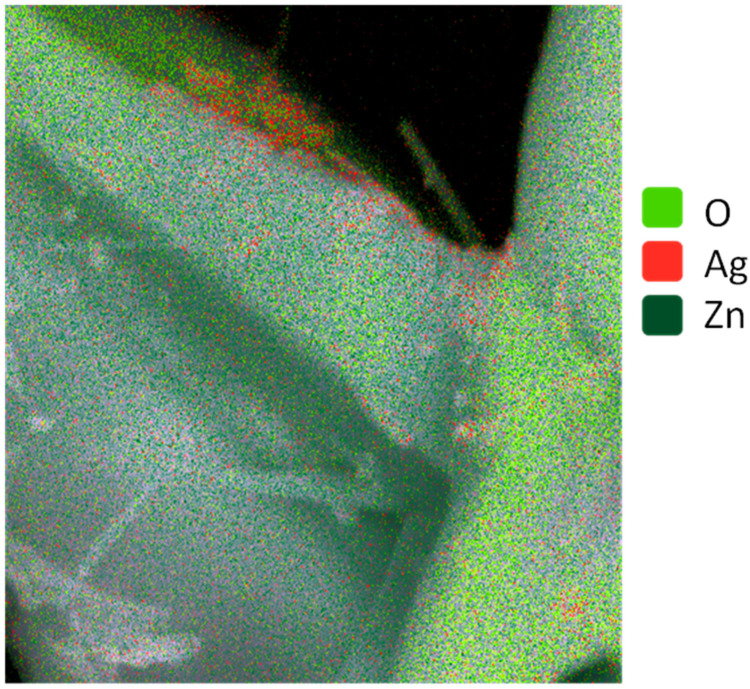
EDX mapping of of typical Ag nanoparticle clusters on ZnO tetrapods.

According to energy-dispersive X-ray (EDX) microanalysis data, Ag content in the studied samples correlates directly with the AgNO_3_ content in the solution (Figure 1). A noticeable Ag content on the surface of ZnO tetrapods is observed at ≥0.025 mM of AgNO_3_ in the solution. The maximum Ag content on the surface of ZnO tetrapods is observed at 1 mM AgNO_3_ in the solution. Due to its chemical inertness, Ag typically exhibits low adhesion to the zinc oxide surface. However, localized charges on the facets and edges of the ZnO tetrapods can facilitate the anchoring of Ag islands. The surface topology of a crystal is often correlated with its electrical relief. From this perspective, the highest density of Ag islands is expected at the edges of ZnO tetrapods. The selective interaction of Ag islands with active local surface sites depends on the island sizes. Island clusters of a large size would interact not with individual electrically active centers but with the surface micro-sites with averaged electrical properties. Larger clusters would be concentrated on the facets (Figure 2 and Figure 3). At low AgNO_3_ content in the solution, discrete Ag islands are formed, while at high content, along with discrete islands, clusters of large Ag particles are observed. Specifically, at 0.75 mM AgNO_3_, branched clusters of Ag islands can be observed (Figure 2a), and upon increasing the concentration to 1 mM, dense accumulations of Ag particles form (Figure 2b).

**Figure 4:**
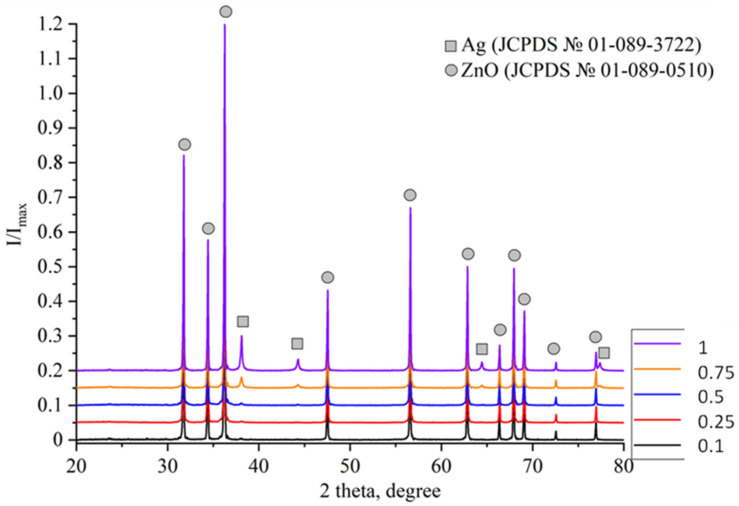
XRD patterns of Ag/ZnO tetrapods with different concentration of Ag nanoparticles. Designations: ■—Ag; ●—ZnO.

X-ray diffraction (XRD) analysis was performed to study the phase composition of samples at different concentrations of Ag nanoparticles (Figure 4), as well as to assess the size of crystallites (coherent scattering regions) and the magnitude of microstresses.

In the sample with the minimum concentration of Ag nanoparticles (correlating to 0.1 mM AgNO_3_ in solution), peaks associated with the ZnO phase (JCPDS No. 01-089-0510) and low-intensity peaks associated with the Ag phase (JCPDS No. 01-089-3722) are observed. The peaks of the ZnO phase were observed in all the studied samples. However, the intensity of the Ag peaks is so low that it was not possible to estimate the content of the Ag phase in the Ag/ZnO tetrapods. A similar picture was observed in the XRD patterns of the samples obtained using the solutions having 0.25 mM and 0.5 mM of AgNO_3_. In the X-ray diffraction pattern of the sample obtained using 0.5 mM AgNO_3_ solution, the Ag (111) and (200) peaks are already distinguishable, but they are still small for estimating the content of the Ag phase. For samples with AgNO_3_ concentrations of 0.75 mM and 1 mM, clearly distinguishable, intense Ag peaks were observed, leading to an assessment of the phase concentration using quantitative phase analysis (Table 1). Quantitative phase analysis was performed using High Scope Plus software v.3.0.5 and the reference intensity ratio (RIR) method. Since the Ag content in the case of samples 0.50 and 0.75 is within the error limits (±1 wt.%) of the method, Table 2 shows the ratios of the intensities of the main peaks of Ag (111) and (200) to the intensity of the maximum peaks in the X-ray diffraction patterns of the samples.

The Ag content in the studied samples, according to RIR data, correlates with the AgNO_3_ content in the solution and is close to EDX data.

**Figure 5:**
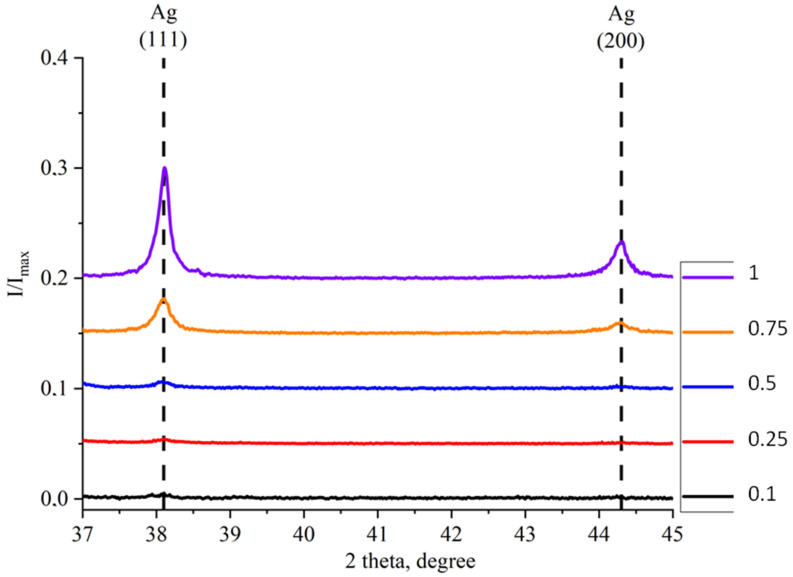
XRD patterns of the 37°–45° region of Ag/ZnO tetrapods containing the main peaks related to Ag nanoparticles.

Table 2 also shows the ratios of the (111) and (200) Ag peak intensities to the maximum intensity in the XRD pattern, clearly demonstrating the increase in Ag peak intensity with increasing Ag nanoparticle concentration (Figure 5).

**Figure 6:**
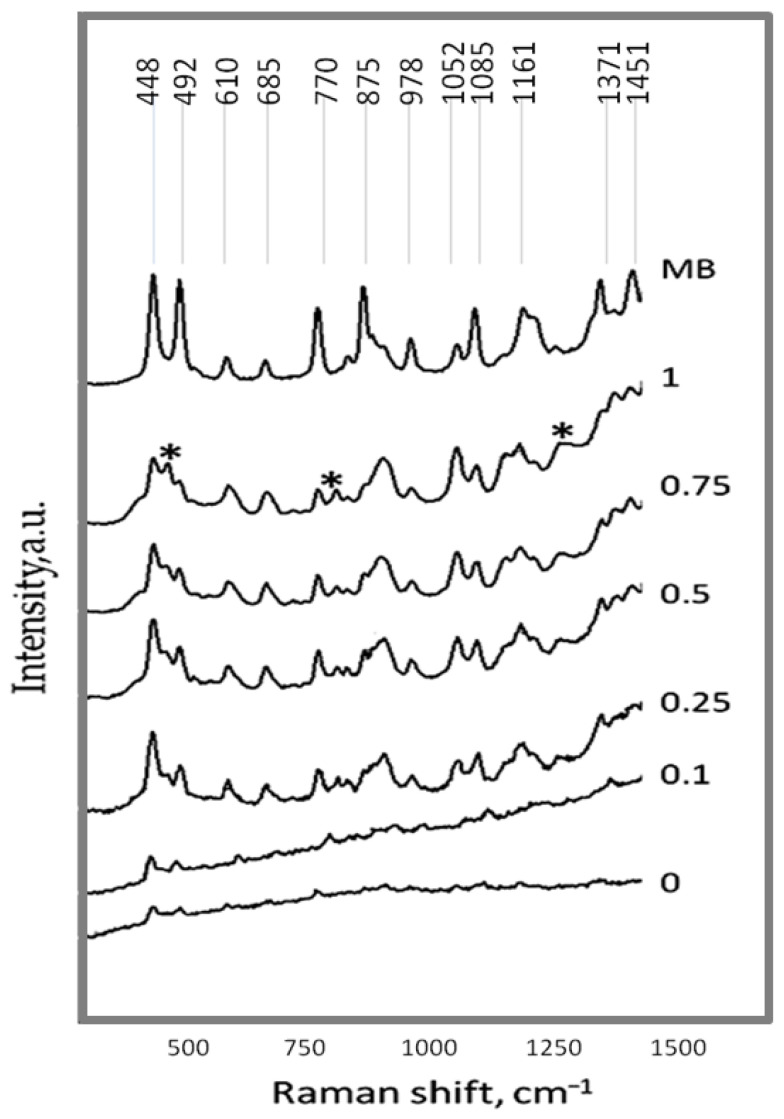
Raman spectra of the MB film deposited on Ag/ZnO tetrapods of various types. For comparison, the Raman spectrum of concentrated MB deposited on a glass substrate is also shown. Asterisks (*) indicate the Raman peaks corresponding to Ag_2_O.

The SERS properties of the Ag/ZnO tetrapods are presented in Figure 6. The Raman spectra of methylene blue (MB) films deposited on Ag/ZnO tetrapods were compared. It can be observed that the spectral dependence of the Raman scattering intensity peaks of MB on the Ag/ZnO tetrapods corresponds to what was obtained earlier in [42]. The modes at 448, 492, 610, 770, 875, 978, 1085, and 1371 cm^−1^ are totally symmetric vibrations, denoted as A modes, whereas the modes at 685, 1052, 1161, and 1451 cm^−1^ are assigned as non-totally symmetric vibration bands, labeled as B mode. Peaks at 487, 818, and 1305 cm^−1^, corresponding to Ag_2_O [43,44] and indicating partial oxidation of silver, are observed. Ag_2_O peaks have the highest intensity in sample type 1, in which the highest Ag concentration is present. It should be noted that the Ag_2_O phase could not be identified on XRD patterns.

The Raman spectra of MB films on sample types 0 and 0.1, which have the lowest Ag concentration, are nearly non-detectable. With an increase in Ag content, the quality of the spectra improves significantly, exhibiting higher resolution and a manifestation of the fine structure of individual intensity peaks. The enhancement factor, determined from the ratio of the intensities of the 451 cm^−1^ peak for MB on Ag/ZnO tetrapods versus pristine ZnO tetrapods, was 5.5–6 for sample types 0.5 and 0.75, and it was approximately 4 for sample type 1. It should be noted that for some peaks, a shift, enhancement, and separation into components were observed, indicating a SERS effect.

### 2.2. Study of Photocatalytic, Piezocatalytic, and Piezo-Photocatalytic Properties of Ag/ZnO Tetrapods

**Figure 7:**
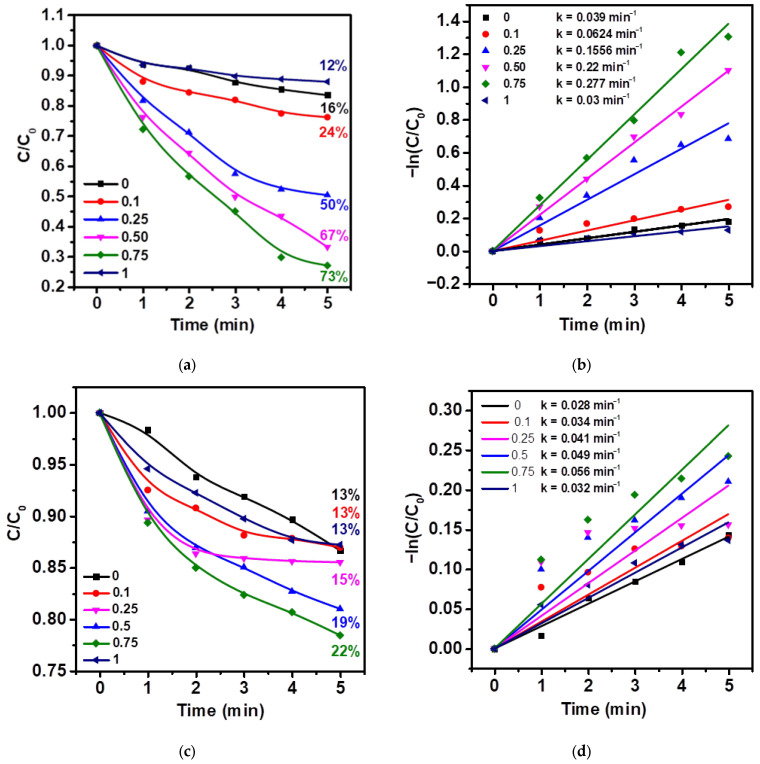
Results of photocatalytic (**a**,**b**) and piezocatalytic (**c**,**d**) degradation of MNZ using pristine ZnO tetrapods (type 0) and Ag-modified samples with various silver contents. (**a**) Temporal variation of the relative concentration $C/C_{0}$ under photocatalytic conditions; (**b**) kinetic analysis based on the pseudo-first-order model. (**c**) Temporal variation of $C/C_{0}$ under piezocatalytic conditions driven by ultrasound (40 kHz, 120 W) in the absence of light; (**d**) corresponding kinetic curves with rate constant calculations.

The photocatalytic properties of ZnO tetrapods and ZnO–Ag composites were investigated under illumination without ultrasonic stimulation (Figure 7a,b).

As shown in Figure 7a, pristine ZnO microtetrapods exhibit low photocatalytic activity, achieving only 16% degradation of MNZ within 5 min of reaction. This poor performance is attributed to the high defect density of the tetrapods caused by the rapid synthesis rate, the fast recombination of photogenerated electron–hole pairs, and the absence of efficient interfacial charge transfer pathways. Modification of ZnO tetrapods with silver leads to a gradual enhancement of photocatalytic activity. With increasing Ag content (type 0.75), the degradation degree reaches 73%. The improvement in activity upon silver incorporation can be explained by at least two factors:

- Formation of a Schottky barrier at the ZnO/Ag interface, where Ag acts as an electron sink, suppressing charge recombination.
- Surface plasmon resonance (SPR) of Ag nanoparticles, which promotes additional electron excitation and the injection of “hot” electrons into the ZnO conduction band.

Kinetic analysis (Figure 7b) confirms that the degradation process follows a pseudo-first-order model. The apparent rate constant k increases more than sevenfold—from 0.039 min^−1^ for type 0 to 0.277 min^−1^ for type 0.75. A further rise in Ag content (type 1) leads to a decrease in k to 0.03 min^−1^, which can be attributed to partial shielding of the ZnO active surface by excess Ag, enhanced back-electron transfer, and aggregation of Ag nanoparticles.

The piezocatalytic activity of the samples was evaluated in the absence of light under ultrasonic irradiation (Figure 7c,d). As seen in Figure 7c, pristine ZnO tetrapods (type 0) show low catalytic efficiency, achieving only 13% degradation of metronidazole after 5 min. A similar result (13%) is observed for type 0.1, indicating that trace amounts of silver do not significantly affect the piezocatalytic activity. With increasing silver content (type 0.25 and 0.50), a gradual rise in degradation efficiency up to 15–19% is observed. The highest piezocatalytic performance (22%) is achieved for type 0.75 (0.75 mmol AgNO_3_). In this case, silver acts as an effective electron acceptor and a conductive “bridge”, facilitating charge extraction from piezopolarized regions and suppressing recombination. However, further Ag loading (type 1) reduces efficiency to 13%. This decline is associated with excessive Ag shielding of surface piezocharges, reduction of the accumulated piezopotential, and formation of metallic percolation paths that accelerate charge recombination. Such behavior is consistent with the SERS data presented in Figure 6.

Similar to photocatalysis, the piezocatalytic efficiency exhibits a nonmonotonic dependence on Ag content, with a well-defined maximum at type 0.75. The kinetic analysis (Figure 7d) corroborates these observations: the rate constant increases from 0.028 min^−1^ (type 0) to 0.056 min^−1^ (type 0.75), followed by a decrease to 0.032 min^−1^ (type 1). This indicates that an optimal Ag amount ensures a balance between interfacial charge transfer and the preservation of effective piezoelectric polarization within the structure.

Overall, both the photocatalytic and piezocatalytic activities of ZnO tetrapods are substantially enhanced after Ag modification, with both processes showing the same trend—activity reaches a maximum at type 0.75 and declines thereafter with further Ag addition. This finding highlights the key electronic and structural role of silver: it is not merely an inert additive but actively participates in interfacial charge transfer processes. Given the pronounced influence of Ag on the electronic processes in ZnO, it is reasonable to hypothesize that the simultaneous application of light and mechanical activation (ultrasound) could induce a combined or synergistic piezo-photocatalytic effect. Such a mechanism involves the coupling of photoinduced excitation and piezoelectric polarization, potentially altering charge transfer dynamics and further improving the catalytic efficiency of the system. To verify this hypothesis, additional experiments on piezo-photocatalytic degradation of metronidazole were conducted, and the results are discussed below.

**Figure 8:**
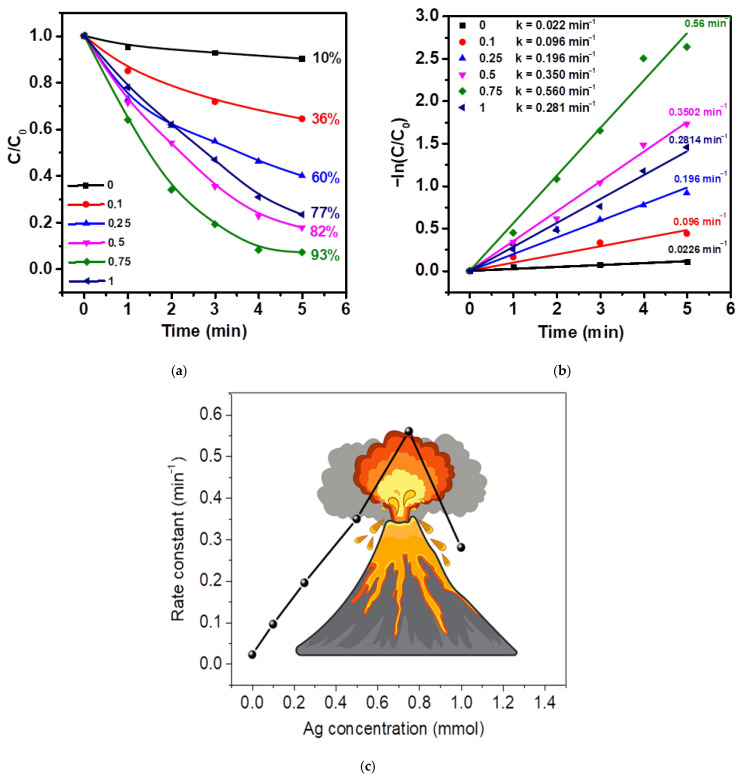
Piezo-photocatalytic degradation of metronidazole over ZnO microtetrapods decorated with different Ag loadings under metal-halide lamp irradiation and ultrasonication (40 kHz, 120 W): (**a**) degradation curves *C*/*C*_0_ vs. time; (**b**) pseudo-first-order kinetics; (**c**) volcano-type dependence of the rate constant on the initial Ag^+^ concentration in the precursor AgNO_3_ solution.

The temporal evolution of MNZ concentration (Figure 8a,b) revealed that pristine ZnO (type 0) exhibits limited catalytic activity, achieving only ~10% degradation after 5 min of reaction. This low efficiency is primarily attributed to the rapid recombination of photogenerated electron–hole pairs and the insufficient separation of charge carriers within the ZnO structure. Despite the inherent piezoelectricity of ZnO, the generated piezoelectric potential under ultrasonic excitation alone was insufficient to markedly accelerate charge transfer in the absence of co-catalysts, leading to restricted oxidative capability.

The deposition of Ag nanoparticles significantly enhanced the degradation efficiency, indicating a pronounced synergistic effect between the plasmonic properties of Ag and the piezoelectric nature of ZnO tetrapods. As the Ag content increased (type 0.1 to type 0.75), the degradation efficiency rose progressively from 36% to 93% within 5 min. This enhancement is attributed to two main factors. First, Ag nanoparticles act as electron traps due to the formation of a Schottky barrier at the Ag/ZnO interface, which suppresses charge recombination and promotes directional charge separation. Second, localized surface plasmon resonance (LSPR) of Ag under visible and near-UV excitation intensifies local electromagnetic fields, thereby increasing light absorption and the generation of hot electrons. Under ultrasonic stimulation, these effects are further amplified by piezoelectric polarization, which induces an internal electric field within ZnO tetrapods that facilitates carrier migration to the surface, where redox reactions occur. The combined piezoelectric and plasmonic effects therefore establish an efficient charge transfer pathway, enhancing the generation of ROS, such as ^•^OH and ^•^O_2_^−^, which are responsible for MNZ degradation.

However, a further increase in Ag (type 1) resulted in a decline in degradation efficiency (82%), indicating the existence of an optimal silver loading. This trend suggests a volcano-type catalytic behavior (Figure 8c), commonly observed in supported metal–semiconductor systems. At higher Ag concentrations, excessive Ag nanoparticles tend to aggregate and shield the ZnO surface, partially blocking active sites and hindering light penetration. The overloading of metallic Ag may also induce electron back-transfer, suppressing the Schottky barrier effect and accelerating recombination. Therefore, an optimal Ag content (type 0.75) achieves a balance between efficient charge separation and maximized surface activation without causing optical shadowing or surface passivation effects.

Kinetic analysis based on the pseudo-first-order model (Figure 8b) supports these observations. The apparent rate constant (k) increased from 0.0026 min^−1^ for pristine ZnO (type 0) to a maximum of 0.56 min^−1^ for type 0.75, representing more than a hundred-fold improvement. This confirms the superior catalytic efficiency under dual-field activation (light + ultrasound) and highlights the strong synergistic interaction between plasmonic excitation and piezo-induced charge polarization. The subsequent decline of k at higher Ag loading (0.256 min^−1^ for type 1) is consistent with decreased availability of the ZnO active surface, confirming that excessive Ag coverage hampers catalytic performance.

The results clearly indicate that the piezo-photocatalytic degradation of metronidazole is governed by efficient charge carrier dynamics enabled by the interfacial engineering of Ag/ZnO tetrapods. The unique 3D morphology of ZnO tetrapods plays an essential role, as their anisotropic structure enables effective mechanical deformation under ultrasound, generating a substantial piezoelectric potential that promotes interfacial charge transfer. Simultaneously, Ag nanoparticles facilitate plasmon-enhanced light harvesting and electron trapping, resulting in sustained ROS production. The observed volcano-type behavior in catalytic activity as a function of Ag loading reflects the interplay between these beneficial effects and detrimental factors such as light shielding and electron back-transfer at high Ag concentrations. These findings demonstrate that the combination of plasmonic Ag nanoparticles and the intrinsic piezoelectricity of ZnO tetrapods yields a highly efficient piezo-photocatalytic system. The optimized Ag content (type 0.75) offers a promising strategy for designing advanced hybrid catalysts for rapid degradation of persistent pharmaceutical pollutants in water.

To quantitatively evaluate the contribution of the combined piezo-photocatalytic process compared to the individual photo- and piezocatalytic activations, the synergistic effect was calculated for the optimal sample (type 0.75). In the literature, the synergistic interaction between two external stimuli (light and ultrasound) is defined as the deviation of the observed reaction rate from the simple sum of their individual contributions [45]. The absolute value of the synergistic effect S is determined by the following expression:

(1) $$S=k_{\mathrm{PPhC}}-(k_{\mathrm{PhC}}+k_{\mathrm{PC}}),$$

where $k_{PPhC}$, $k_{PhC}$, and $k_{PC}$ are the rate constants of piezo-photocatalysis, photocatalysis, and piezocatalysis, respectively. Substituting the experimental values *k*_PPhC_ = 0.560 min^−1^, *k*_PhC_ = 0.277 min^−1^, and *k*_PC_ = 0.056 min^−1^, we obtain

(2) $$S=0.560-\left(0.277+0.056\right)=0.227\;\min^{-1}.$$

The positive value of the synergistic parameter (S>0) indicates that the simultaneous action of light and ultrasound does not merely result in an additive combination of photocatalytic and piezocatalytic effects but instead gives rise to a new, enhanced charge transfer mechanism that cannot be achieved under isolated activation conditions.

This conclusion is further supported by the synergistic enhancement coefficient (*SE*), defined as

(3) $$SE=\frac{k_{ppHC}h}{k_{phHC}+k_{PC}},$$

which corresponds to a 68% increase in efficiency relative to the additive contribution of the two independent processes.

Despite the obtained kinetic dependencies, these results alone do not reveal the fundamental origins of the observed differences in catalytic activity, nor do they elucidate the nature of the underlying electronic processes governing these behaviors. In particular, the question remains open as to why the type 0.75 sample exhibits optimal silver concentration, ensuring the highest reaction rates for both photo- and piezocatalysis, whereas a further increase in Ag content leads to a decline in efficiency.

To clarify the microscopic origins of this behavior and to determine the role of the defect subsystem in charge transfer, additional studies of the electronic defect structure were performed using electron paramagnetic resonance (EPR) and photoluminescence (PL) spectroscopy. For comparative analysis, two representative samples were selected: the pristine ZnO tetrapods (type 0) and the optimized Ag-modified sample (type 0.75) exhibiting the best catalytic performance.

**Figure 9:**
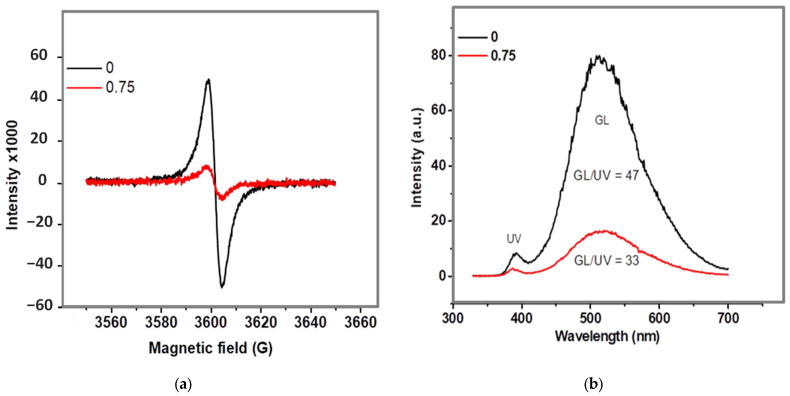
Electron paramagnetic resonance (EPR) (**a**) and photoluminescence (PL) (**b**) spectra of ZnO tetrapods before and after surface modification with 0.75 mM of AgNO_3_.

This approach made it possible to assess whether the enhanced activity of the modified sample is associated with (i) changes in the concentration of oxygen vacancies, (ii) redistribution of surface charge states, (iii) formation of interfacial transitions at the ZnO/Ag boundary, or (iv) suppression of defect-mediated recombination processes. The EPR spectra (Figure 9) provide direct insight into nature and charge states of point defects in the ZnO tetrapod structure and allow tracing their transformations upon silver modification.

In the EPR spectra of pristine ZnO, a single-component signal with a g-factor of approximately g ≈ 2.00 is observed, which is characteristic of paramagnetic centers associated with oxygen vacancies $V_{O}^{\cdot }$ containing a localized unpaired electron (S = 1/2) and exhibiting an *s*-like electron density near the crystal surface [46,47]. The dominance of this signal and the absence of contributions from deeper defect levels indicate the prevailing role of surface donor-type oxygen vacancies in nanostructured ZnO tetrapods. This finding is consistent with previously reported EPR data for oxide nanostructures, where surface $V_{O}^{\cdot }$ centers are recognized as the principal active species [48].

It is noteworthy that no resonance lines corresponding to zinc vacancies ($V_{Zn}$)—which typically exhibit a more pronounced *g*-tensor and spectral anisotropy [47]—or their complexes are detected. This is attributed to the fact that zinc vacancies in ZnO act as acceptor-type defects, which in the nanoscale regime exist predominantly in a diamagnetic state ($V_{Zn}^{2-}$) and are therefore EPR-silent [49]. After surface modification with silver, the intensity of the EPR signal decreases significantly, while both the resonance field position and the *g*-factor remain unchanged. This clearly indicates that the defects are not eliminated but rather undergo a change in their charge state due to interfacial electron redistribution within the ZnO–Ag heterostructure. According to the metal–n-type semiconductor contact theory [48] and experimental studies on Ag/ZnO interfacial transitions [50], the formation of a heterojunction leads to electron transfer from surface oxygen vacancies to Ag until thermodynamic equilibrium of the Fermi levels is established. As a result, vacancies transform from the paramagnetic $V_{O}^{\cdot }$ (EPR-active) state to the diamagnetic $V_{O}^{\cdot \cdot }$ configuration, leading to a reduction in EPR signal intensity. Additionally, Holston et al. [51] provided further evidence that, in addition to oxygen vacancies, complex defects such as zinc–oxygen divacancies ($V_{Zn}$–$V_{O}$) can also exist at or near the ZnO surface, whose ionization states depend on local charge redistribution. These centers are typically electron-compensated and remain EPR-inactive unless externally excited. The photoluminescence spectra (Figure 9b) corroborate the EPR findings and reveal the functional role of defects within the electronic structure of the material. The PL spectra of pristine ZnO exhibit two characteristic emission bands: (i) a near-band-edge UV emission (~380 nm), associated with interband and excitonic transitions, and (ii) a broad green luminescence (GL) band (500–600 nm), typical of defect-related recombination through oxygen vacancy levels and their complexes [52].

The high intensity ratio $I_{GL}/I_{UV}=47$ in pristine ZnO indicates a dominant contribution of defect-related states to the recombination processes, consistent with the high concentration of $V_{O}^{\cdot }$ centers detected by EPR. After Ag modification, a significant suppression of the GL band ($I_{GL}/I_{UV}=33$) is observed, which is interpreted as a reduction of radiative recombination through defect levels. Moreover, the narrowing of the GL band indicates the suppression of deep-trap transitions, reflecting reduced electron localization at defect sites in the presence of silver.

Taken together, the EPR and PL data demonstrate that the formation of the ZnO–Ag interface leads to charge redistribution within the near-surface region of ZnO. The transition $V_{O}^{\cdot }\to V_{O}^{\cdot \cdot }$ and partial compensation of associated defects result in realignment of the surface potential and redistribution of electronic density, forming a space-charge region and Fermi-level alignment. This is manifested as a decrease in EPR intensity and quenching of defect-related luminescence. Analogous charge-redistribution processes and defect-induced interfacial restructuring have previously been described in detail for plasmonic Au/TiO_2_ and Au/TiO_2−x_ systems [53,54,55]. According to these studies, in the absence of illumination, electrons migrate from donor centers (Ti^3+^/V_O_) of the reduced oxide to the metal until Fermi-level equilibration is reached and the effective Schottky barrier is lowered, leading to partial compensation of vacancies and the formation of a depletion layer. The electron-coupled interfacial states formed in this process play a key role in mediating interfacial charge transfer.

Furthermore, oxygen vacancies act not only as local donor defects but also as mediators of interfacial charge transfer. According to contemporary models of defect-assisted carrier transport, localized states associated with $V_{O}^{\cdot }$ can serve as electron-bridging centers, enabling tunneling or stepwise transfer of electrons between energy levels. This defect-mediated transport mechanism has been confirmed in several ZnO-based heterostructures and plays a crucial role in interfacial electron transfer processes [56].

**Figure 10:**
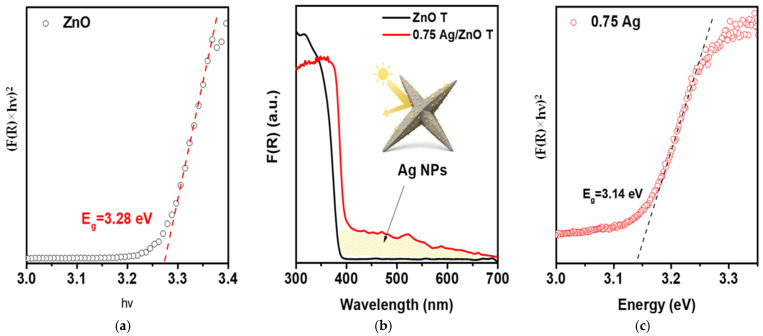
Optical properties of pristine ZnO tetrapods and Ag-decorated ZnO (0.75 mmol AgNO_3_). (**a**,**c**) Tauc plots for pristine and 0.75 Ag-modified ZnO T. (**b**) Diffuse reflectance spectra (Kubelka–Munk function) of ZnO and 0.75 Ag/ZnO, demonstrating the strong plasmon-induced absorption enhancement in the visible region (yellow region), associated with localized surface plasmon resonance (LSPR) of Ag nanoparticles anchored on ZnO tetrapod facets.

To validate these conclusions, the optical properties of the samples were analyzed (Figure 10).

As seen from the diffuse reflectance spectra (Figure 10b), the introduction of Ag leads to a pronounced increase in light absorption within the visible region (400–800 nm). This effect is unequivocally attributed to the excitation of localized surface plasmon resonance (LSPR) of silver nanoparticles with sizes of 50–200 nm (as confirmed by the SEM data presented in the article). Due to the broad size distribution and island-like morphology of the Ag deposits, the plasmon band becomes significantly broadened, resulting in a smooth increase in absorption without a well-defined maximum. The enhanced absorption in the 400–600 nm range reflects the generation of “hot” electrons in Ag and the amplification of the local electromagnetic field at the ZnO/Ag interface.

The Tauc analysis (Figure 10a,c) demonstrates that the fundamental optical band gap remains nearly unchanged; however, a slight reduction (from 3.28 to 3.14 eV) is observed. This decrease is not related to bulk modifications of ZnO but instead has an interfacial origin and arises from charge redistribution at the ZnO/Ag boundary, accompanied by the formation of a space-charge region and the emergence of additional surface electronic states. The partial depopulation of ZnO surface donor levels facilitates electron transfer between the oxide and silver, creating favorable conditions for the formation of a stable ZnO/Ag heterojunction. These interfacial states, located closer to the conduction band edge, promote charge transfer from ZnO to Ag and contribute to the more efficient separation of photogenerated electron–hole pairs.

**Figure 11:**
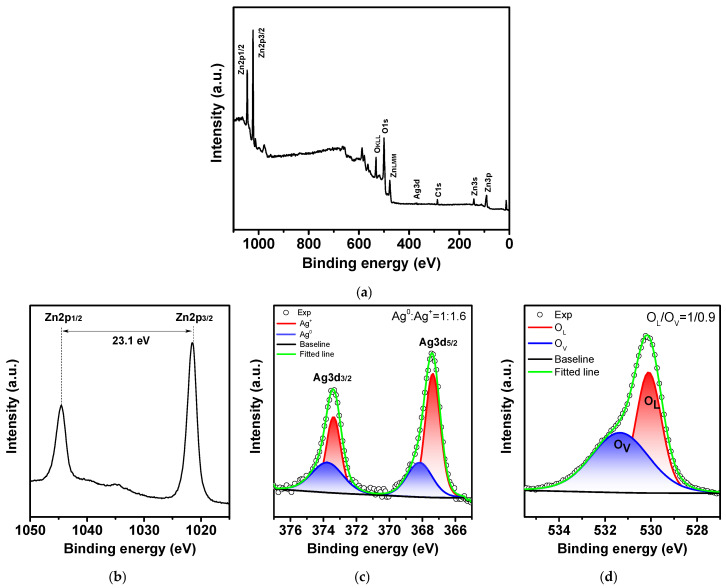
Panoramic XPS spectra of Ag/ZnO–0.75 (**a**); (**b**) XPS spectra Zn 2p, typical for Zn^2+^; (**c**) XPS spectra Ag 3d with components Ag^0^ and Ag^+^, characterizing the metal core and the surface oxide layer; (**d**) XPS spectra O 1s, including lattice and defective oxygen.

X-ray photoelectron spectroscopy (XPS) was used to analyze the surface composition and chemical states of the optimal sample, Ag/ZnO-0.75. In a survey spectrum (Figure 11a), the main elements of the system (Zn, O, Ag) are clearly identified, with no impurity-related lines observed, which confirms the high chemical purity of the composite. Deconvolution of Zn 2p-spectra (Figure 11b) reveals two well-resolved peaks, Zn 2p3/2 and Zn 2p1/2, with characteristic spin-orbit splitting at 23.1 eV, which corresponds to Zn^2+^ in a ZnO crystal lattice. The absence of any shifts or additional components in the spectrum indicates that no Zn reduction or formation of secondary phases occurs, confirming that the ZnO structure is preserved after modification with Ag.

In the Ag region (Figure 11c), two pairs of peaks are observed, corresponding to Ag^0^ and Ag^+^. The Ag^0^–Ag^+^ ratio ≈ 1:1.6 indicates that the oxidized form of Ag predominates in the surface layer. However, X-ray diffraction detects only metallic silver, indicating an absence of crystalline Ag_2_O or AgO phases. Such an ambiguity can be explained by the surface-sensitive nature of the XPS method (analysis depth 3–5 nm): after photodeposition, silver nanoparticles develop a thin amorphous AgO_x_ oxide layer that is not detectable by XRD but is readily observed by XPS.

The presence of the oxide component is also confirmed by Raman spectroscopy, which detects vibrational features characteristic for Ag-O bonds. These results are consistent with a core–shell model, in which a metallic Ag^0^ core, responsible for the plasmonic properties, is coated with a thin amorphous AgO_x_ layer, governing the electron-transfer processes.

The O 1s spectrum (Figure 11d) can be well fitted with two main components: a low-energy one, O_L_ (~530.2 eV), corresponding to lattice oxygen in Zn-O, and a higher-energy one, O_V_ (~531.5–532 eV), associated with surface oxygen vacancies and adsorbed reactive O species. For 0.75 sample, the O_L_/O_v_ ratio is approximately 0.9, indicating a high concentration of surface oxygen vacancies—these are the key donor sites involved in charge transfer, which is fully consistent with the EPR and photoluminescence data.

**Figure 12:**
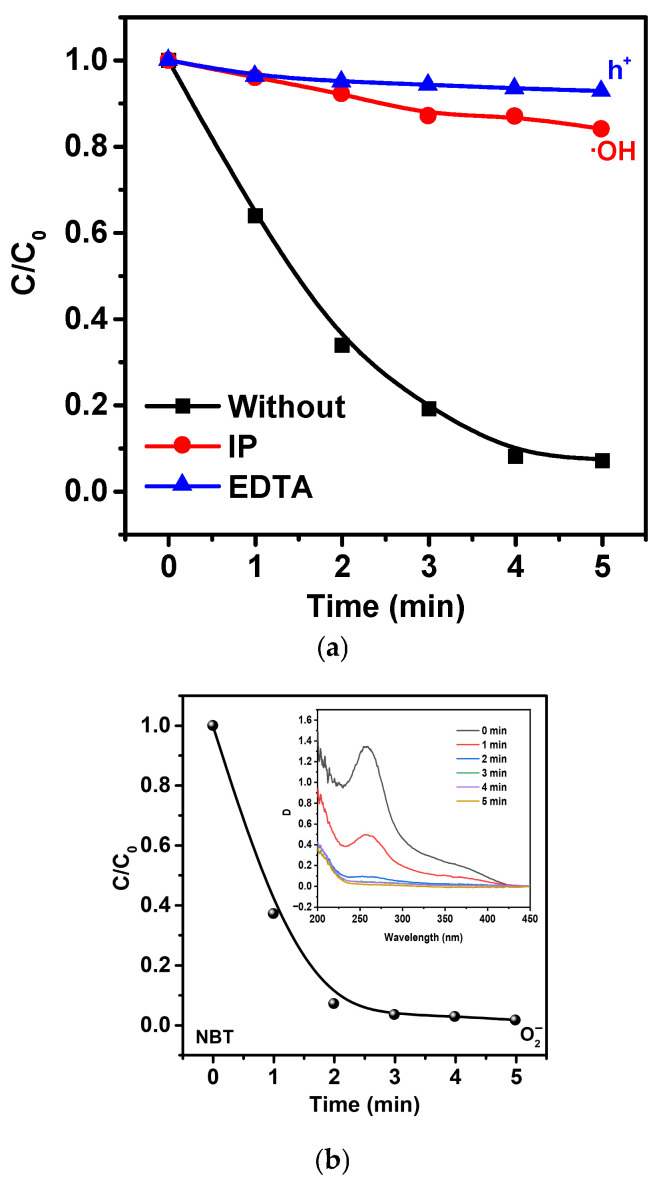
ROS trapping experiments for the 0.75 catalyst under piezo-photocatalytic conditions. (**a**) Effect of selective scavengers on the degradation kinetics (IPA (^•^OH scavenger) and EDTA-2Na (h^+^ scavenger)). (**b**) Detection of superoxide radicals using nitro blue tetrazolium (NBT).

To study the mechanisms responsible for the enhanced piezo-photocatalytic activity of Ag/ZnO microtetrapods, it is crucial to identify which ROS participate in the process and to determine their relative contributions. Figure 12a shows the effect of different scavengers on the degradation kinetics of the target pollutant over the 0.75 catalyst under piezo-photocatalytic conditions.

The addition of isopropanol (IP), a well-established ^•^OH scavenger, leads to a pronounced suppression of the degradation rate, reducing the conversion after 5 min from nearly complete degradation (≈93%) to ~16%. This behavior indicates that hydroxyl radicals (^•^OH) constitute one of the dominant oxidative species in the system.

The influence of EDTA, a strong hole (h^+^) scavenger, is even more pronounced: the degradation efficiency drops to ~92% inhibition relative to the unquenched experiment. The strong suppression of catalytic activity in the presence of EDTA confirms that photogenerated holes (h^+^) play a primary role as oxidizing agents, consistent with efficient charge separation at the ZnO/Ag interface. Figure 12b presents the results of superoxide detection using nitro blue tetrazolium (NBT), which selectively reacts with ^•^O_2_^−^ to form reduced formazan products. A rapid decrease in the absorbance of NBT (inset) is observed during the first minutes of reaction, indicating active formation of superoxide radicals. The time-dependent decay of NBT concentration (main panel) follows a characteristic pseudo-first-order profile, confirming efficient generation of ^•^O_2_^−^ under piezo-photocatalytic excitation.

Under ultrasonic excitation, the ZnO microtetrapods undergo anisotropic mechanical deformation, which generates a localized piezoelectric field. These internal electric fields promote efficient separation of photogenerated charge carriers and direct the transport of electrons and holes toward different surface regions. Simultaneously, the plasmonic excitation of Ag nanoparticles produces hot electrons that are injected into the conduction band of ZnO, further enhancing the electron flow induced by mechanical deformation.

ZnO + hν + ultrasound → e_CB_^−^ + h_VB_^+^ Ag + hν → Ag(LSPR) → Ag(e_hot_^−^) Ag(e_hot_^−^) → e_CB_(ZnO)^−^ + Ag (4)

Conduction-band electrons, further accelerated by the piezoelectric field, reduce molecular oxygen to form superoxide radicals (^•^O_2_^−^), whereas holes, retained at the surface by the opposite piezocharges, oxidize water or hydroxide ions to generate hydroxyl radicals (^•^OH).

e_CB_(ZnO)^−^ + O_2_ → ^•^O_2_^−^ h_VB_^+^ + H_2_O → ^•^OH + H^+^ h_VB_^+^ + OH^−^ → ^•^OH (5)

Both radical species, together with the direct oxidation of MNZ by holes (h^+^), collectively ensure the rapid degradation of the pollutant molecule.

^•^OH + MNZ → intermediate products → CO_2_ + H_2_O ^•^O_2_^−^ + MNZ → oxidized intermediates h_VB_^+^ + MNZ → MNZ^+^ → further oxidation (6)

An essential contribution to these processes is provided by the Ag/ZnO Schottky-type interface: in the dark, it attracts electrons toward the Ag phase, whereas under illumination it facilitates the reverse plasmon-induced injection of electrons back into ZnO, thereby lowering the charge transfer barrier and enhancing carrier separation under the action of the piezoelectric fields. The synergy between piezoelectric deformation, plasmonic excitation of Ag, and defect-modulated charge transfer is fully consistent with the identified dominant roles of h^+^, ^•^OH и ^•^O_2_^−^ and accounts for the exceptionally high activity of the 0.75 sample.

In Refs. [31,32], long-term stability tests and evaluations of application potential were carried out. After five photocatalytic cycles, the ZnO tetrapod samples retained their degradation efficiency and exhibited stable structural characteristics.

To assess the efficiency and prospects of zinc oxide (ZnO) microtetrapods decorated with plasmonic Ag nanoparticles developed in this study, a systematic comparative analysis was performed against previously reported Ag–ZnO photocatalysts (see Table 3).

Analysis of published plasmonic and heterostructured ZnO–Ag systems shows that high degradation efficiencies (90–100%) are typically achieved either under intense illumination (xenon or halogen lamps of 250–500 W), under strong ultrasonic treatment (120–200 W, 40–45 kHz), or when using readily oxidizable dyes at low concentrations.

In most studies (Table 3), complete degradation of organic dyes is achieved within 25–240 min, but only at considerable energy consumption and with a high loading of catalytic nanostructures. For example, ZnO-T/Ag_2_O under UV irradiation (365 nm, 50 W) combined with 200 W ultrasound provides 100% degradation in 2 min, while requiring a catalyst loading of 2 g/L. Systems such as ZnO/AgI, ZnO/Ag_3_PO_4_, and Ag_2_S/ZnO require 250–300 W xenon lamps and 120–200 W ultrasound to reach a comparable performance within 30–120 min.

Compared with these examples from the literature, our Ag/ZnO microtetrapod system achieves comparable or even higher degradation efficiency while requiring a significantly lower catalyst concentration, reduced optical power, and substantially milder excitation conditions. This performance is achieved through the combination of three factors: (i) the highly developed 3D geometry of the tetrapods, which enables efficient deformation and enhanced piezoelectric fields; (ii) plasmon-active Ag nanoparticles that broaden the optical absorption spectrum and provide hot-electron transfer; and (iii) a defect-modulated ZnO–Ag interface that reduces the Schottky barrier and facilitates interfacial carrier transport. This results in performance comparable to the best literature analogs, but at a significantly lower energy input, which highlights the efficiency of the proposed approach.

## 3. Materials and Methods

Two methods were used to synthesize ZnO microtetrapods: high-temperature pyrolysis [6] and using atmospheric-pressure microwave nitrogen plasma [31].

Zinc oxide tetrapods were synthesized by high-temperature pyrolysis. Metal Zn granules ~5 mm in diameter (99.999% purity, trace metals basis) produced by Alfa Aesar were used as a precursor. Ash-free cellulose filters served as a carbon source. The synthesis was performed in corundum crucibles at 1050 °C under isothermal conditions for 50 min. The inner diameter of the corundum crucibles was 40 mm. The diameter of the air inlet window was 2 mm. The ZnO tetrapods reached several tens of micrometers in size.

Microwave plasma synthesis of ZnO was carried out using a 2.45 GHz waveguide plasmatron operating at atmospheric pressure. The system comprised a magnetron source (up to 3 kW CW power), a WR-340 rectangular waveguide with a water-cooled load, and a quartz discharge tube (30 mm ID) mounted perpendicularly to the waveguide. High-purity nitrogen (99.998%) was supplied at ~10 L·min^−1^, carrying zinc powder (30–40 µm) through the plasma region. A tangential gas inlet generated a vortex flow, which stabilized the discharge and prevented local wall overheating. Part of the synthesized ZnO powder exited the reactor, while a white deposit near the outlet was collected for analysis. The plasma core temperature reached 5000–6000 K (n_e_ ~ 10^13^ cm^−3^, T_e_ ~ 1 eV), decreasing sharply to ≈950 K near the inner wall close to the tube exit. The tetrapods reached several micrometers in size.

According to the results of the experiments, microtetrapods of maximum size were selected. Silver photo-deposition was carried out via photochemical reduction of Ag^+^ ions on the surface of ZnO microtetrapods. To synthesize the ZnO–Ag(x) nanocomposites, a weighed portion of 20 mg of ZnO tetrapods was placed into a 50 mL glass beaker (GG-17), followed by the addition of 1 mL of freshly prepared aqueous AgNO_3_ solution containing different amounts of silver precursor: 0.10, 0.25, 0.50, 0.75, and 1.00 mmol. The suspension was thoroughly dispersed using ultrasonic agitation for 5 min to ensure uniform particle distribution.

Photo-deposition was performed under irradiation from a high-pressure mercury lamp (Philips, Amsterdam, The Netherlands, 250 W) emitting in the UV–visible range, with the distance between the suspension surface and the lamp fixed at 10 cm. The samples were irradiated for 60 min. After illumination, the solid product was separated and repeatedly washed—first with deionized water (3 × 5 mL) to remove residual nitrate ions and then with ethanol (2 × 5 mL) to prevent aggregation and accelerate drying. The resulting powders were dried at room temperature for 12 h. The obtained samples were labeled according to the introduced silver concentration as types 0.1, 0.25, 0.5, 0.75, and 1, respectively. For comparison, a control ZnO sample (type 0) was prepared under identical conditions but without the addition of AgNO_3_.

Microscopic studies were conducted using a JCM-6000 (JEOL, Tokyo, Japan) scanning electron microscope (SEM) equipped with an energy-dispersive X-ray (EDX) microanalyzer. X-ray diffraction (XRD) patterns were obtained on an Empyrean diffractometer (PANalytical, Almelo, The Netherlands) in Bragg–Brentano geometry with CuKα radiation (λ = 1.54 Å).

Raman spectra were recorded on an Ntegra Spectra (NT-MDT) facility (Zelenograd, Moscow, Russia) at a diode laser wavelength of 532 nm, 20 mW power, and a beam spot of ~5 microns in diameter. A 0.1 mM solution from MB was prepared to measure SERS spectra. For the SERS property investigation of the Ag/ZnO tetrapods, a special substrate was fabricated. Ag/ZnO tetrapods were dispersed in ethanol. On the next step, a drop of the solution was applied to the surface of the glass substrate and evaporated; this procedure was repeated 5 times. The EPR/ESR spectra of the studied samples were obtained using an EMX Plus X-band electron paramagnetic resonance spectrometer (Bruker, Billerica, MA, USA). For all samples, spectra were recorded over a wide range of magnetic fields (0–6000 G) to examine the presence of all possible signals. Photoluminescence (PL) spectra were recorded on a SM 2203 spectrofluorometer (SOL Instruments, Minsk, Belarus). The experimental details of the optical characterization procedure are provided in our previous work [32]. To determine the chemical composition of the Ag/ZnO tetrapods via X-ray photoelectron spectroscopy (XPS), a SPECS XPS spectrometer (Specs, Berlin, Germany) equipped with an Al anode was used. The choice of anode material was made to avoid interference from Auger lines in the useful signal. Spectra were recorded in the binding energy range from 0 to 1200 eV. The calibration of binding energies was performed using the C-C line of the C1s spectrum (E binding = 284.8 eV).

In this experiment, metronidazole (MNZ) (5 mg/L) was used as a model water pollutant to evaluate the piezo-, photo-, and piezo-photocatalytic properties of the synthesized catalysts. The piezocatalytic activity of the catalysts was assessed by their ability to degrade MNZ under ultrasonic irradiation, photocatalytic activity under simulated solar light irradiation, and piezo-photocatalytic activity under simultaneous exposure to both light and ultrasound.

The piezo-photocatalytic experiments were carried out in a 2 L ultrasonic bath with a power of 120 W and a frequency of 40 kHz (Shenzhen Fanying Ultrasonic Technology Co., Ltd., Shenzhen, China), using a 75 W metal-halide lamp (Osram, Munich, Germany) as the light source, without any optical filters. The experiments were conducted in a 50 mL glass beaker. A constant temperature was maintained at 26 °C through ventilation and thermometer monitoring. To initiate the reaction, 20 mg of photocatalyst was added to 20 mL of MNZ aqueous solution.

Prior to experiments, the beaker was maintained in darkness for 30 min to establish an adsorption–desorption equilibrium. The suspension was subjected to ultrasonic treatment to degas the photocatalyst before conducting the experiment. The light source was positioned 10 cm above the beaker. At 1 min intervals, 5 mL probes were extracted and filtered through Nylon syringe filters with pore sizes of 220 nm to separate the microparticles. The concentration of MNZ was quantified using an SF-2000 spectrophotometer (OKB Spectr LLC, Saint-Petersburg, Russia) based on the characteristic MNZ absorption peak at 320 nm wavelength. After measuring, the solution was poured back into the reactor, and the process continued. To confirm the role of the catalyst in the degradation of MB, control experiments were carried out under the same conditions but without the addition of the catalyst. The control experiments included exposure to ultrasound and light separately.

ROS trapping tests were carried out for the most active catalyst, under the same piezo-photocatalytic conditions as the main experiment. To evaluate the contribution of individual reactive species, 1 mmol of each scavenger was added to the reaction mixture: isopropanol (IP, 1 mmol)—hydroxyl radical (^•^OH) scavenger; EDTA-2Na (1 mmol)—hole (h^+^) scavenger. After adding the scavenger, the suspension was stirred for 5 min at room temperature, followed by piezo-photocatalytic testing (ultrasound + light). The pollutant concentration was monitored spectrophotometrically and compared with the control experiment without scavengers.

The generation of ^•^O_2_^−^ was also investigated using a similar piezo-photocatalytic procedure. A solution of nitro blue tetrazolium chloride (NBT) with a concentration of 20 mg/L was used as the test solution, and approximately 4 mL of the NBT solution was taken at specific time intervals for analysis using spectrophotometry to determine the optical density at 259 nm.

## 4. Conclusions

In this study, zinc oxide (ZnO) microtetrapods with a well-defined 3D architecture were synthesized via high-temperature pyrolysis and using atmospheric-pressure microwave nitrogen plasma. According to the results of the experiments, microtetrapods of maximum size were selected (several tens of micrometers in size). Subsequent photochemical deposition of silver nanoparticles (with tunable sizes ranging from 50 to 200 nm, depending on precursor concentration) yielded Ag/ZnO heterostructures with controlled surface coverage. Systematic evaluation revealed that the catalytic performance of these materials is highly sensitive to silver loading. The optimal Ag content (0.75 mmol of AgNO_3_) resulted in maximal photocatalytic, piezocatalytic, and piezo-photocatalytic efficiencies. Under combined light irradiation and ultrasonic stimulation, the 0.75 Ag/ZnO composite achieved 93% degradation of metronidazole (MNZ) within 5 min, corresponding to an apparent rate constant of 0.56 min^−1^—over 24 times higher than that of pristine ZnO tetrapods.

This enhanced activity is attributed to the synergistic coupling between the plasmonic excitation of silver nanoparticles and the intrinsic piezoelectric polarization of ZnO microtetrapods. Structural and spectroscopic analyses (XRD, EPR, PL) confirmed that the introduction of silver not only forms Schottky barriers for improved charge transfer but also suppresses defect-mediated recombination through redistribution of electronic density at the ZnO–Ag interface.

Importantly, the volcano-type dependence of catalytic behavior on silver content underscores the critical balance between enhanced interfacial charge dynamics and adverse effects such as surface shielding at higher metal loadings. These findings highlight the relevance of structural morphology and nanoscale engineering in designing advanced catalytic systems. The demonstrated plasmon-enhanced piezo-photocatalytic strategy offers a promising route for the rapid and efficient degradation of persistent organic contaminants in water.

## Figures and Tables

**Table 1:** Phase ratio in samples.

Type of Sample	ZnO, %	Ag, %
0.1	~100	~0
0.25	~100	~0
0.50	~99.9	~0.1
0.75	98.5	1.5
1	97.0	3.0

**Table 2:** Ratio of the intensities of the main peaks of Ag (111) and (200) to the intensity of the maximum peaks in the X-ray diffraction patterns of the samples.

		Type of Sample				
		0.1	0.25	0.5	0.75	1
I/I_max_	Ag (111)	0.00446	0.00519	0.00618	0.03154	0.10058
	Ag (200)	0.00124	0.00245	0.00143	0.00986	0.03304

**Table 3:** Comparative summary of piezo-photocatalytic properties of Ag-ZnO-based catalysts in the degradation of organic pollutants.

Catalyst	Light Source	Source of Mechanical Impact	Catalyst Loading	Pollutant (Type, Conc.)	Reaction Time	Efficiency, % Rate Const	Ref.
Ag/ZnO microtetrapods	metal-halide lamp (75 W)	US (120 W, 40 kHz)	20 mg	MNZ (5 mg/L, 20 mL)	5 min	93%/ 0.56 min^−1^	This work
ZnO-T/Ag_2_O	UV light (50 W, 365 nm)	US (200 W)	2 g/L	MB (5 mg/L, 50 mL)	2 min	100%/ 1.51 min^−1^	[57]
ZnO-T/Ag	Spherical Xe lamp (500 W)	US probe (200 W)	2 g/L	MO (5 mg/L, 100 mL)	25 min	100% 0.17 min^−1^	[29]
ZnO/AgS nanowire	Xenon lamp (300 W) with a cut-off filter (λ ≤ 400 nm)	Magnetic stirrer (500 rpm)	On Ni foam (2 cm × 2 cm, thickness: 1.5 mm)	RhB (2.5 mg/L, 30 mL)	30 min	96.6% 0.24542 min^−1^	[58]
ZnO-T/Ag_8_S	Halogen lamp (20 V, 250 W)	US (50 W)	-	RhB (1 × 10^−3^ M, 100 mL)	120 min	93.11% 0.0102 min^−1^	[59]
ZnO/AgI nanoflower	Xenon lamp (250 W, λ > 400 nm, I ~ 100 mW/cm^2^)	US (40 kHz, 120 W)	20 mg	RhB (10 mg/L, 100 mL)	40 min	97.2%	[60]
Ag_2_S/ZnO nanowire	Solar illumination (I = 80 mW/cm^−2)^	US (45 kHz)	-	MB (1 mg/L, 100 mL)	240 min	100%	[61]
PVDF@Ag- ZnO/Au nanofibers	Xenon lamp (300 W)	US (200 W, 40 kHz)	-	RhB (10 mg/L, 50 mL)	120 min	98.8%/ 0.04 min^−1^	[62]
Ag_3_PO_4_/ZnO nanowire	Xenon lamp (300 W) with a cut-off filter (λ ≤ 400 nm)	US (50 W, 40 kHz)	30 mg	MB	30 min	98.16%/ 0.1341 min^−1^	[63]
Cloisite 30B/ZnO/Ag_2_O	UV lamp (15 W, 254 nm)	US (24 kHz, 180 W)	0.5 g/L	MB (5 mg/L)	50 min	98.43%	[64]

## Data Availability

The original contributions presented in this study are included in the article. Further inquiries can be directed to the corresponding authors.

## References

[1] Bryche J.-F., Vega M., Tempez A., Brulé T., Carlier T., Moreau J., Chaigneau M., Charette P.G., Canva M. (2022). Spatially-Localized Functionalization on Nanostructured Surfaces for Enhanced Plasmonic Sensing Efficacy. Nanomaterials.

[2] Fomin V.M., Marquardt O. (2023). Topology- and Geometry-Controlled Functionalization of Nanostructured Metamaterials. Appl. Sci..

[3] Fu D., Reif J. (2021). 3D DNA Nanostructures: The Nanoscale Architect. Appl. Sci..

[4] Colombelli A., Lospinoso D., Rella R., Manera M.G. (2022). Shape Modulation of Plasmonic Nanostructures by Unconventional Lithographic Technique. Nanomaterials.

[5] Shen S., Gao M., Ban R., Chen H., Wang X., Qian L., Li J., Yang Z. (2018). Spatially-Controllable Hot Spots for Plasmon-Enhanced Second-Harmonic Generation in AgNP-ZnO Nanocavity Arrays. Nanomaterials.

[6] Krasnova V.V., Muslimov A.E., Lavrikov A.S., Zadorozhnaya L.A., Orudzhev F.F., Gulakhmedov R.R., Kanevsky V.M. (2024). Characterization and Photocatalytic Properties of ZnO Tetrapods Synthesized by High-Temperature Pyrolysis. Crystallogr. Rep..

[7] Terracciano M., Račkauskas S., Falanga A.P., Martino S., Chianese G., Greco F., Piccialli G., Viscardi G., De Stefano L., Oliviero G. (2023). ZnO Tetrapods for Label-Free Optical Biosensing: Physicochemical Characterization and Functionalization Strategies. Int. J. Mol. Sci..

[8] Escobedo-Morales A., Aranda-García R.J., Chigo-Anota E., Pérez-Centeno A., Méndez-Blas A., Arana-Toro C.G. (2016). ZnO Micro- and Nanostructures Obtained by Thermal Oxidation: Microstructure, Morphogenesis, Optical, and Photoluminescence Properties. Crystals.

[9] Orudzhev F., Gadzhiev M., Abdulkerimov M., Muslimov A., Krasnova V., Il’ichev M., Kanevsky V. (2025). Plasma-Assisted Synthesis of TiO_2_/ZnO Heterocomposite Microparticles: Phase Composition, Surface Chemistry, and Photocatalytic Performance. Molecules.

[10] Lamberti A. (2018). ZnO- and TiO_2_-Based Nanostructures. Nanomaterials.

[11] Pujara A., Sharma R., Samriti, Bechelany M., Mishra Y.K., Prakash J. (2025). Novel zinc oxide 3D tetrapod nano-microstructures: Recent progress in synthesis, modification and tailoring of optical properties for photocatalytic applications. Mater. Adv..

[12] Ushanov V.I., Eremeev S.V., Silkin V.M., Chaldyshev V.V. (2024). Plasmon Resonance in a System of Bi Nanoparticles Embedded into (Al,Ga)As Matrix. Nanomaterials.

[13] Liu R., Zhan D., Wang D., Han C., Fu Q., Zhu H., Mao Z., Liu Z.-Q. (2023). Surface Plasmon Resonance Effect of Noble Metal (Ag and Au) Nanoparticles on BiVO_4_ for Photoelectrochemical Water Splitting. Inorganics.

[14] Jiang P., Wang K., Liu W., Song Y., Zheng R., Chen L., Su B. (2024). Hot Electrons Induced by Localized Surface Plasmon Resonance in Ag/g-C_3_N_4_ Schottky Junction for Photothermal Catalytic CO_2_ Reduction. Polymers.

[15] Busila M., Musat V., Alexandru P., Romanitan C., Brincoveanu O., Tucureanu V., Mihalache I., Iancu A.-V., Dediu V. (2023). Antibacterial and Photocatalytic Activity of ZnO/Au and ZnO/Ag Nanocomposites. Int. J. Mol. Sci..

[16] Chen P.-T., Lu Y.-C., Tangsuwanjinda S., Chung R.-J., Sakthivel R., Cheng H.-M. (2022). Irradiation-Induced Synthesis of Ag/ZnO Nanostructures as Surface-Enhanced Raman Scattering Sensors for Sensitive Detection of the Pesticide Acetamiprid. Sensors.

[17] Orudzhev F., Sobola D., Ramazanov S., Částková K., Papež N., Selimov D.A., Abdurakhmanov M., Shuaibov A., Rabadanova A., Gulakhmedov R. (2023). Piezo-Enhanced Photocatalytic Activity of the Electrospun Fibrous Magnetic PVDF/BiFeO_3_ Membrane. Polymers.

[18] Lv H., Wang P., Lv Y., Dong L., Li L., Xu M., Fu L., Yue B., Yu D. (2025). Piezo-Photocatalytic Degradation of Ciprofloxacin Based on Flexible BiVO_4_ PVDF Nanofibers Membrane. Catalysts.

[19] He J., Dong C., Chen X., Cai H., Chen X., Jiang X., Zhang Y., Peng A., Badsha M.A.H. (2023). Review of Piezocatalysis and Piezo-Assisted Photocatalysis in Environmental Engineering. Crystals.

[20] Selimov D.A., Sobola D., Shuaibov A., Schubert R., Gulakhmedov R., Rabadanova A., Orudzhev F. (2025). Coupling Photocatalysis and Piezocatalysis in PVDF/ZnO Nanofiber Composites for Efficient Dye Degradation. Chim. Techno Acta.

[21] Selimov D.A., Rabadanova A.A., Shuaibov A.O., Magomedova A.G., Abdurakhmanov M.G., Gulakhmedov R.R., Orudzhev F.F. (2024). Piezo-Enhanced Photocatalytic Activity of BaTiO_3_-Doped Polyvinylidene Fluoride Nanofibers. Kinet. Catal..

[22] Shuaibov A.O., Abdurakhmanov M.G., Magomedova A.G., Omelyanchik A., Salnikov V., Aga-Tagieva S., Orudzhev F.F. (2024). Sonophotocatalytic Degradation of Methylene Blue with Magnetically Separable Zn-Doped-CoFe_2_O_4_/α-Fe_2_O_3_ Heterostructures. J. Mater. Sci. Mater. Electron..

[23] Orudzhev F.F., Magomedova A.G., Kurnosenko S.A., Beklemyshev V.E., Li W., Wang C., Zvereva I.A. (2025). Tuning of Photocatalytic and Piezophotocatalytic Activity of Bi_3_TiNbO_9_ via Synthesis-Controlled Surface Defect Engineering. Molecules.

[24] Bhadwal N., Ben Mrad R., Behdinan K. (2023). Review of Zinc Oxide Piezoelectric Nanogenerators: Piezoelectric Properties, Composite Structures and Power Output. Sensors.

[25] Polewczyk V., Magrin Maffei R., Vinai G., Lo Cicero M., Prato S., Capaldo P., Dal Zilio S., di Bona A., Paolicelli G., Mescola A. (2021). ZnO Thin Films Growth Optimization for Piezoelectric Application. Sensors.

[26] Ye W., Zi-Chang Z., Shaikh A. (2018). First-principles investigation of size-dependent piezoelectric properties of bare ZnO and ZnO/MgO core-shell nanowires. Superlattices Microstruct..

[27] Su X., Zhao X., Cui C., Xi N., Zhang X.L., Liu H., Yu X., Sang Y. (2022). Influence of Wurtzite ZnO Morphology on Piezophototronic Effect in Photocatalysis. Catalysts.

[28] Muslimov A., Orudzhev F., Gadzhiev M., Selimov D., Tyuftyaev A., Kanevsky V. (2022). Facile synthesis of Ti/TiN/TiON/TiO_2_ composite particles for plasmon-enhanced solar photocatalytic decomposition of methylene blue. Coatings.

[29] Zhang L., Zhu D., He H., Wang Q., Xing L., Xue X. (2017). Enhanced piezo/solar photocatalytic activity of Ag/ZnO nanotetrapods arising from the coupling of surface plasmon resonance and piezophototronic effect. J. Phys. Chem. Solids.

[30] Reis A.T., Costa C., Fraga S. (2023). Editorial of Special Issue: The Toxicity of Nanomaterials and Legacy Contaminants: Risks to the Environment and Human Health. Int. J. Mol. Sci..

[31] Muslimov A., Antipov S., Gadzhiev M., Ulyankina A., Krasnova V., Lavrikov A., Kanevsky V. (2023). Oxidation of Zinc Microparticles by Microwave Plasma to Form Effective Solar-Sensitive Photocatalysts. Appl. Sci..

[32] Orudzhev F., Muslimov A., Selimov D., Gulakhmedov R.R., Lavrikov A., Kanevsky V., Gasimov R., Krasnova V., Sobola D. (2023). Oxygen Vacancies and Surface Wettability: Key Factors in Activating and Enhancing the Solar Photocatalytic Activity of ZnO Tetrapods. Int. J. Mol. Sci..

[33] Ning X., Hao A., Cao Y., Hu J., Xie J., Jia D. (2020). Effective promoting piezocatalytic property of zinc oxide for degradation of organic pollutants and insight into piezocatalytic mechanism. J. Colloid Interface Sci..

[34] Liao H., Fang J., Wang J., Long X., Zhang I.Y., Huang R. (2024). Effective Degradation of Metronidazole through Electrochemical Activation of Peroxymonosulfate: Mechanistic Insights and Implications. Energies.

[35] Maldonado Domínguez S.M., Barrera-Díaz C.E., Balderas Hernández P., Amado-Piña D., Torres-Blancas T., Roa-Morales G. (2025). Metronidazole Electro-Oxidation Degradation on a Pilot Scale. Catalysts.

[36] Lanzky P.F., HallingSorensen B. (1997). The toxic effect of the antibiotic metronidazole on aquatic organisms. Chemosphere.

[37] Bendesky A., Menendez D., Ostrosky-Wegman P. (2002). Is metronidazole carcinogenic?. Mutat. Res. Rev. Mutat. Res..

[38] Sun J., Chu R., Khan Z.U.H. (2023). A Theoretical Study on the Degradation Mechanism, Kinetics, and Ecotoxicity of Metronidazole (MNZ) in •OH- and SO_4_^•−^-Assisted Advanced Oxidation Processes. Toxics.

[39] Hama Aziz K.H., Mustafa F.S., Karim M.A.H., Hama S. (2025). Pharmaceutical Pollution in the Aquatic Environment: Advanced Oxidation Processes as Efficient Treatment Approaches: A Review. Mater. Adv..

[40] Luan J., Li Z., Yao Y., Wang J., Hao L. (2025). Metronidazole Degradation via Visible Light-Driven Z-Scheme BiTmDySbO_7_/BiEuO_3_ Heterojunction Photocatalyst. Sustainability.

[41] Shuvo M.S.H., Putul R.A., Hossain K.S., Masum S.M., Molla M.A.I. (2024). Photocatalytic Removal of Metronidazole Antibiotics from Water Using Novel Ag-N-SnO_2_ Nanohybrid Material. Toxics.

[42] Dutta Roy S., Ghosh M., Chowdhury J. (2015). Adsorptive parameters and influence of hot geometries on the SER(R) S spectra of methylene blue molecules adsorbed on gold nanocolloidal particles. J. Raman Spectrosc..

[43] Fauzia M.A., Khan A., Parveen A., Almohammedi A.A. (2024). Enhanced antibacterial and visible light driven photocatalytic activity of graphene oxide mediated Ag_2_O nanocomposites. Opt. Quantum Electron..

[44] Khubezhov S., Ponkratova E., Kuzmichev A., Maleeva K., Larin A., Karsakova E. (2024). Fastand scalable fabrication of Ag/TiO_2_ nanostructured substrates for enhanced plasmonic sensing and photocatalytic applications. Appl. Surf. Sci..

[45] Rabadanova A., Selimov D., Gulakhmedov R.R., Magomedova A.G., Ronoh K., Částková K., Sobola D., Kaspar P., Shuaibov A., Abdurakhmanov M.G. (2025). Piezophotocatalytic Activity of PVDF/Fe_3_O_4_ Nanofibers: Effect of Ultrasound Frequency and Light Source on the Decomposition of Methylene Blue. ACS Omega.

[46] Janotti A., Van de Walle C.G. (2009). Fundamentals of zinc oxide as a semiconductor. Rep. Prog. Phys..

[47] Hoffmann K., Hahn D. (1974). Electron Spin Resonance of Lattice Defects in Zinc Oxide. Phys. Status Solidi A.

[48] Tuomisto F., Saarinen K., Look D.C., Farlow G.C. (2005). Introduction and recovery of point defects in electron-irradiated ZnO. Phys. Rev. B.

[49] Vlasenko L.S., Watkins G.D. (2005). Optical detection of electron paramagnetic resonance in room-temperature electron-irradiated ZnO. Phys. Rev. B.

[50] McCluskey M.D., Jokela S.J. (2009). Defects in ZnO. J. Appl. Phys..

[51] Sze S.M., Ng K.K. (2007). Physics of Semiconductor Devices.

[52] Trang T.N.Q., Phan T.B., Nam N.D., Thu V.T.H. (2020). In Situ Charge Transfer at the Ag@ZnO Photoelectrochemical Interface toward the High Photocatalytic Performance of H_2_ Evolution and RhB Degradation. ACS Appl. Mater. Interfaces.

[53] Lin L., Feng X., Lan D., Chen Y., Zhong Q., Liu C., Cheng Y., Qi R., Ge J., Yu C. (2020). Coupling Effect of Au Nanoparticles with the Oxygen Vacancies of TiO_2−x_ for Enhanced Charge Transfer. J. Phys. Chem. C.

[54] Li H., Wang S., Tang J., Xie H., Ma J., Chi H., Li C. (2023). Roles of oxygen vacancies in surface plasmon resonance photoelectrocatalytic water oxidation. Cell Rep. Phys. Sci..

[55] Cheng J., Cui J., Liang J., Khan S.N., Jia J. (2025). Synergy between oxygen vacancies and SPR effect in of Bi@Vo-Bi2O3 for efficient photocatalytic performance under visible light. Chemsuschem..

[56] Holston M.S., Golden E.M., Kananen B.E., McClory J.W., Giles N.C., Halliburton L.E. (2016). Identification of the zinc-oxygen divacancy in ZnO crystals. J. Appl. Phys..

[57] Sun C., Fu Y., Wang Q., Xing L., Liu B., Xue X. (2016). Ultrafast piezo-photocatalytic degradation of organic pollutions by Ag2O/tetrapod-ZnO nanostructures under ultrasonic/UV exposure. RSC Adv..

[58] Qu S., Lan J., Yan J., Khan I., Żak M., Zheng Y., Ma M., Wang Z., Guo S., Huang S. (2025). ZnO/Ag_2_S heterogeneous nanowire arrays on porous nickel foam: Synergistic piezo-photocatalytic enhancement for high-efficiency environmental remediation. Surf. Interfaces.

[59] Venugopal G., Thangavel S., Vasudevan V., Zoltán K. (2020). Efficient visible-light piezophototronic activity of ZnO-Ag8S hybrid for degradation of organic dye molecule. J. Phys. Chem. Solids.

[60] Liu J., Chen J., Wu Z., Zhu K., Wang J., Li Z., Tai G., Liu X., Lu S. (2021). Enhanced visible-light photocatalytic performances of ZnO through loading AgI and coupling piezo-photocatalysis. J. Alloys Compd..

[61] Zhang Y., Liu C., Zhu G., Huang X., Liu W., Hu W., Song M., He W., Liu J., Zhai J. (2017). Piezotronic-effect-enhanced Ag_2_S/ZnO photocatalyst for organic dye degradation. RSC Adv..

[62] Li F., Shan B., Zhao X., Ji C., Li Z., Yu J., Xu S., Jiao Y., Zhang C., Man B. (2022). Plasmonic enhanced piezoelectric photoresponse with flexible PVDF@Ag-ZnO/Au composite nanofiber membranes. Opt. Express.

[63] Yu Y., Yao B., He Y. (2020). Piezo-enhanced photodegradation of organic pollutants on Ag_3_PO_4_/ZnO nanowires using visible light and ultrasonic. Appl. Surf. Sci..

[64] Foroutan R., Peighambardoust S.J., Boffito D.C., Ramavandi B. (2022). Sono-Photocatalytic Activity of Cloisite 30B/ZnO/Ag_2_O Nanocomposite for the Simultaneous Degradation of Crystal Violet and Methylene Blue Dyes in Aqueous Media. Nanomaterials.

